# Identification of novel candidate genes for 46,XY disorders of sex development (DSD) using a C57BL/6J-Y^*POS*^ mouse model

**DOI:** 10.1186/s13293-018-0167-9

**Published:** 2018-01-30

**Authors:** Hayk Barseghyan, Aleisha Symon, Mariam Zadikyan, Miguel Almalvez, Eva E. Segura, Ascia Eskin, Matthew S. Bramble, Valerie A. Arboleda, Ruth Baxter, Stanley F. Nelson, Emmanuèle C. Délot, Vincent Harley, Eric Vilain

**Affiliations:** 1 Center for Genetic Medicine Research, Children’s Research Institute, Children’s National Health System, Washington, DC, 20010 USA; 2 Department of Human Genetics, David Geffen School of Medicine, University of California, Los Angeles, CA 90095 USA; 3 Department of Pediatrics, David Geffen School of Medicine, University of California, Los Angeles, CA 90095 USA; 4 Department of Brain and Gender, Hudson Institute of Medical Research, Clayton, VIC 3168 Australia

**Keywords:** Disorders of sex development, 46,XY DSD, Undervirilization, C57BL/6J mouse, RNA-Seq, Exome, Gonadal dysgenesis

## Abstract

**Background:**

Disorders of sex development (DSD) have an estimated frequency of 0.5% of live births encompassing a variety of urogenital anomalies ranging from mild hypospadias to a discrepancy between sex chromosomes and external genitalia. In order to identify the underlying genetic etiology, we had performed exome sequencing in a subset of DSD cases with 46,XY karyotype and were able to identify the causative genetic variant in 35% of cases. While the genetic etiology was not ascertained in more than half of the cases, a large number of variants of unknown clinical significance (VUS) were identified in those exomes.

**Methods:**

To investigate the relevance of these VUS in regards to the patient’s phenotype, we utilized a mouse model in which the presence of a Y chromosome from the *poschiavinus* strain (Y^*POS*^) on a C57BL/6J (B6) background results in XY undervirilization and sex reversal, a phenotype characteristic to a large subset of human 46,XY DSD cases. We assessed gene expression differences between B6-Y^*B6*^ and undervirilized B6-Y^*POS*^ gonads at E11.5 and identified 515 differentially expressed genes (308 underexpressed and 207 overexpressed in B6-Y^*POS*^ males).

**Results:**

We identified 15 novel candidate genes potentially involved in 46,XY DSD pathogenesis by filtering the list of human VUS-carrying genes provided by exome sequencing with the list of differentially expressed genes from B6-Y^*POS*^ mouse model. Additionally, we identified that 7 of the 15 candidate genes were significantly underexpressed in the XY gonads of mice with suppressed *Sox9* expression in Sertoli cells suggesting that some of the candidate genes may be downstream of a well-known sex determining gene, *Sox9.*

**Conclusion:**

The use of a DSD-specific animal model improves variant interpretation by correlating human sequence variants with transcriptome variation.

**Electronic supplementary material:**

The online version of this article (10.1186/s13293-018-0167-9) contains supplementary material, which is available to authorized users.

## Background

Human sex development is dictated by the inheritance of either an X or Y chromosome from the father to offspring. The male sex determination step starts with the expression of a Y-chromosome-encoded transcription factor *SRY* (sex-determining region Y) in the bipotential gonad, initiating a cascade of molecular and cellular events leading to testicular organogenesis [1]. In the absence of the Y chromosome, female-specific pathways are initiated for proper ovarian development [2]. Sex differentiation then occurs, mostly under the influence of testicular (e.g., testosterone, AMH) or ovarian (e.g., estradiol) hormones or transcription factors (e.g., *COUP-TFII*) that further differentiate the body into typical male or female structures, including both internal and external genitalia [3, 4]. Anomalies in hormonal exposure and/or gene mutations disrupting sex development pathways lead to disorders of sex development (DSD) [5–7], defined as “congenital conditions in which development of chromosomal, gonadal, or anatomic sex is atypical” [8]. The umbrella term DSD encompasses conditions ranging from mild hypospadias (abnormal location of the meatus) to discrepancy between sex chromosomes and external genital phenotype (formerly known as sex reversal, either complete or with ambiguous genitalia). DSDs are estimated to affect up to 0.5% of the population [9]. The birth of a child with a DSD may be highly stressful for families, bringing uncertainty in regard to the child’s future psychosexual development and clinical management [8, 10, 11].

At present, a specific molecular diagnosis is identified at variable rates in different DSD conditions, and gonadal dysgenesis cases are arguably the most difficult to diagnose. The majority (80–90%) of isolated 46,XX testicular DSD are explained by *SRY* translocations, but only a minority (~ 10%) of ovotesticular DSD in 46,XX individuals are [12]. Copy number variants of the *SOX9* and *SOX3* gene regions are a well-established etiology but only explain a few cases [12]. More recently, a single nucleotide variant in *NR5A1* (nuclear receptor subfamily 5 group A member 1) gene resulting in p.Arg92Trp amino acid change has been associated with 46,XX testicular (and ovotesticular) DSD [13, 14]. The majority of cases of ovarian dysgenesis occur in individuals with an abnormal sex chromosome complement, most commonly 45,X (Turner syndrome), but the advent of next-generation sequencing has recently identified many autosomal genes implicated in determination and maintenance of the ovarian fate. They affect various processes, in particular DNA repair, replication, and stability, but explain a minority of cases [15, 16]. Among 46,XY DSD cases with gonadal dysgenesis, about 15% each are due to *SRY*, *NR5A1*, and *MAP3K1* (mitogen-activated protein kinase kinase kinase 1), and rare cases have been attributed to mutations in other genes such as *SOX9* (SRY-box9), *NR0B1* (nuclear receptor subfamily 0 group B member 1), or *FGFR2* (fibroblast growth factor receptor 2) [17, 18]. Nevertheless, collectively, the genetic etiology is still not identified in greater than 50% of DSD patients, suggesting the existence of a number of unknown sex-determining genes. We endeavored to identify novel candidate genes for 46,XY gonadal dysgenesis.

Next-generation sequencing has become instrumental in DSD diagnosis, including clinical exome sequencing and gene panels [17, 19–21] with high diagnostic rates reported for known DSD genes. In a cohort of 46,XY DSD patients, we established a diagnosis in approximately 1/3 of cases [22], similar to rates for other rare disorders [23, 24]. Another 15% of the exomes in the cohort contained variants of unknown significance (VUS) in known DSD genes that could not be validated as pathogenic but were reported to the referring clinicians to orient further endocrine or imaging testing toward a definitive diagnosis (the variants were termed as “actionable VUS”). Half of the cases from our cohort remained undiagnosed but contained hundreds of VUS that provide an opportunity for identification of novel etiologies for DSD. Here, we utilize an animal model of DSD with gonadal dysgenesis and undervirilization [25, 26] to identify a group of genes that were misexpressed during disrupted testis development. This list was cross-referenced with the list of VUS from 46,XY DSD patients to predict which VUS might be causative in cases where exome sequencing did not result in a definitive diagnosis. We show that the identified 15 novel candidate genes contain a VUS identified in 46,XY DSD cases and are expressed at the time of sex development in a sex-differential manner. In addition, we show that the expression of many of these genes in the developing male gonads is dependent on the known sex-determining gene *Sox9*.

## Methods

### Exome sequencing and analysis

DNA was isolated from peripheral blood using Gentra Puregene Blood Kit (Qiagen, USA) or saliva collected using ORAgene ORG-500 (DNAgenoteck, Canada). Sequencing libraries and exome capture was done for each sample following manufacturer’s protocols for SureSelect All Exon 50 Mb capture kit (Agilent Technologies) and Nextera Rapid Capture (Illumina, USA). Sequencing was performed on an Illumina HiSeq2500 as 50-100 bp paired-end run at the UCLA Clinical Genomics Center.

The sequence reads, FASTQ files, were aligned to the human reference genome (GRCh37/hg19 Feb. 2009 assembly) using BWA (Burrows-Wheeler Alignment tool) [27] and Novoalign (novocraft.com). The output BAM files were sorted and merged, and PCR duplicates were removed using Picard. INDEL (insertion and deletion) realignment and recalibration was performed using Genome Analysis Tool Kit (GATK) (broadinstitute.org). Both single-nucleotide variants (SNVs) and small INDELs were called within the Ensembl coding exonic intervals ± 2 bp using GATK’s Unified Genotyper, then recalibrated and filtered using GATK variant-quality score recalibration and variant filtration tools. All high-quality variants were annotated using SNP&Variation Suite and VarSeq—variant filtration and annotation software (Golden Helix, USA). All variants were filtered by a minor allele frequency (MAF) of < 1% and intersected with the DSD gene list to identify mutations in known DSD genes. The list is comprised of a primary gene list of well-annotated genes involved in sex determination and differentiation [17], as well as a secondary list of genes that are more loosely associated with sex development, e.g., their OMIM (Online Mendelian Inheritance of Man) description contains sex development keywords.

The variants identified by exome sequencing were classified into causative or likely causative variants following the recommendations of the American College of Medical Genetics and Genomics [28]. All other variants with minor allele frequency below 1% were classified as variants of unknown significance (VUS). To assess previously unreported missense variants, we used two in silico algorithms SIFT [29] and PolyPhen [30] to predict the pathogenicity of a missense variant based on conservation of the amino acid across species, the physical characteristics of the altered amino acid, and the possible impact on protein structure and function. All variants with low quality scores were validated by Sanger sequencing [31].

### Animal care and dissections

The C57BL/6J and C57BL/6J-Y^*POS*^ animals were housed at the UCLA Animal Care Facility following the guidelines of the University of California, Los Angeles, Division of Laboratory Animal Medicine. All experiments were approved by the Institutional Animal Care and Research Committees of UCLA. Wild-type C57BL/6J males and females used for breeding were purchased from the Jackson Laboratory (Bar Harbor, USA), which is fully accredited by the American Association for Accreditation of Laboratory Animal Care.

We have previously identified a 1.5-Mb congenic region on chromosome 11 that confers 80% protection from B6-Y^*POS*^ sex reversal in the heterozygous state (B6-110h-Y^*POS*^) and complete protection in the homozygous state (B6-110H-Y^*POS*^) [25]. This protective region allows for continual maintenance of subfertile *poschiavinus* male mice as a breeding colony, with an option of generating unprotected B6-Y^*POS*^ males by mating heterozygous B6-110h-Y^*POS*^ males with wild-type (WT) B6 females. Overnight mating was performed using either the wild-type (WT) B6 or B6-110h-Y^*POS*^ (protected from sex reversal) males and WT B6 females. Dissections were performed at E11.5; the gonads were separated from the mesonephros and placed in RNA stabilizing solution RNA*later* (Ambion). DNA was extracted from the rest of the embryos for genotyping. Chromosomal sex was determined using a single primer pair for X-linked *Smc*-x gene (330 bp) and the Y-linked *Smc*-y gene (301 bp) (forward: 5′CCGCTGCCAAATTCTTTGG3′; reverse: 5′TGAAGCTTTTGGCTTTGAG3′). The presence of the Y^*POS*^ chromosome was determined by a SNP between Y^B6^ and Y^*POS*^ Sry gene using the primer sets 5′TGAATGCATTTATGGTGTGGTC3′; 5′AGCTTTGCTGGTTTTTGGAGTA3′. Immomix Red (Bioline, UK). Presence or absence of the 110 h protective region in B6-Y^*POS*^ males was checked by Sanger sequencing of two regions 11-10 and 11-11 containing SNP rs27019103 (5′AAAGTGTGCTTCCCAGGAGA3′; 5′CCTCTCCCTCAACCCCTAAG3′) and SNP rs28240850 (5′CCACAGCTGGAGGTAGGGTA3′; 5′CCTAAGATGCCATGGGAAGA3′) respectively [25].

Total RNA was isolated from combined embryonic gonadal tissue (50–70 gonads per group) using Qiagen RNeasy Kit (Qiagen) following manufacturer’s guidelines. RNA quality was assessed by Agilent 2100 Bioanalyzer (Agilent Technologies). All samples were required to have RNA integrity scores (RIN) greater than 8.

Experiments on *AmhCre Sox9floxflox* mice were carried out in strict adherence with the recommendations in the Australian code of practice for the care and use of animals for scientific purposes from the National Health and Medical Research Council.

E13.5 gonads were separated from the mesonephros and total RNA was isolated using the RNeasy Kit (Qiagen), as described in Rahmoun et al. [32]. Embryos were genotyped, and the RNA from the six gonads was pooled into wild-type XY, XX, or XY *AmhCre Sox9floxflox* (*Sox9* knockout). This was repeated in three biological replicates; protocols are detailed in Rahmoun et al. [32].

### RNA sequencing and expression analysis

RNA from each sample was submitted to the UCLA Neuroscience Genomic Core (UNGC) for library preparation and sequencing. Library preparation was performed using TruSeq Stranded Total RNA kit (Illumina) with Poly-A selection following manufacturer’s guidelines. Sequencing was performed on HiSeq 2500 (Illumina) with 69 bp paired-end run on a rapid flowcell capable of generating 150 M reads per lane. Four samples were multiplexed and sequenced over two rapid lanes with each sample receiving approximately 75 million reads with > 85% map rate.

The generated sequencing reads were aligned to the mouse genome, version mm10 with STAR [33]. Transcript abundance was assessed by Cufflinks (v2.1.1) [34], using a GTF file based on Ensembl mouse NCBI37. Differential expression analysis was based on fold change differences greater than 1.5 between the groups being compared. Differentially expressed genes were split into two categories: underexpressed and overexpressed in B6-Y^*POS*^ males. Both categories were separately subjected to pathway enrichment analysis using Gene Ontology Consortium [35].

To analyze the RNA from *AmhCre Sox9floxflox* XY gonads and wild-type XY and XX gonads, libraries were generated using the NuGEN Mondrian Technology and SPIA amplification methodology, and the data was processed and aligned to the mouse genome (Ensembl version 38.77) as described by Rahmoun et al. [32]. To eliminate composition biases, the trimmed mean of *M* values (TMM) method was used for normalization between the samples [36]. The adjusted *P* value of 0.05 was used to assess which genes were differentially expressed between XY and *AmhCre Sox9floxflox* XY (*Sox9* KO). Graphs of gene expression were made using GraphPad Prism.

### Quantitative PCR

Reverse transcription of RNA to cDNA was performed using Tetro cDNA Synthesis Kit (Bioline, UK) following manufacturer’s protocol. The primer sequences used are detailed in Additional file 1: Table S1. Primers were designed using autoprime software (autoprime.de) and spanned exon-exon junctions for optimal RNA quantification. cDNA was quantified using QuBit HS (Invitrogen) for double-stranded DNA, and a total of 3 ng of cDNA was used per sample for amplification. qPCR was carried out in duplicates using SensiFAST™ SYBR No-ROX Kit (Bioline, UK) by DNA Engine Opticon® 2 real-time PCR detection system (BioRad, USA). Reaction conditions were as follows: 95 °C for 10 min, then 40 cycles of 95 °C for 15 s, 60–64 °C (see Additional file 1: Table S1) for 10 s, and 72 °C for 15 s. Data was analyzed via Opticon Monitor Software (BioRad). Standard curves were generated from a mix of cDNA of all tested samples with five iterations of 1:4 dilutions. Average cycle threshold values (Ct) for each gene/sample were determined based on two replicates. Complementary DNA amounts were estimated based on Ct values and linear equation *y* = *mx* + *b* (where *y* is the Ct value, *m* is the slope, *x* is the cDNA amount, and *b* is the intercept).

### Immunohistochemistry

*Fbln2* expression in the embryonic gonads at E12.5 was assessed using immunohistochemistry following the experimental design of Wilhelm et al. [37], using the anti-*Fbln2* rabbit polyclonal, sc-30176 (Santa Cruz Biotechnology) antibody. Topro (Invitrogen) was used to counterstain nuclei. All images were taken on a Zeiss LSM 510 Meta confocal microscope.

For the assessment of Sox9 and laminin expression in wild-type and *AmhCre Sox9 floxflox* gonads at E13.5, embryos were fixed overnight in 4% paraformaldehyde (PFA) at 4 °C, then washed three times in 1× PBS. The embryos were processed and embedded into paraffin, cut at 5 μm, and mounted onto slides. The slides were baked at 60 °C (30 min), deparaffinized using three washes of xylene, and hydrated using three washes of 100% ethanol, then distilled water and 1× PBS. Antigen retrieval was performed by microwaving slides (on high) in 10 mM sodium citrate (pH = 6.0) for 20 min. Sections were then blocked for 30 min with 5% normal donkey serum, and stained overnight at 4 °C with primary antibodies for anti-Sox9 rabbit polyclonal (1:400) and anti-Laminin rabbit polyclonal (1:100). Sections were washed three times in 1× PBS with 0.1% Tween20 (1× PBST) and incubated with the fluorescent-conjugated secondary antibodies, Donkey anti-rabbit AlexaFluor488 (Thermo Fisher, 1 μg/mL), for 1 h at room temp. Sections were washed three times in 1× PBST, then incubated in 0.1% Sudan Black in 70% EtOH for 5 min to quench background autofluorescence. Lastly, sections were washed three times in 1× PBST, counterstained using DAPI, then washed three times in 1× PBS and mounted using Dako Fluorescent Mounting Medium (Dako). Sections were imaged using fluorescence microscopy (Olympus Corp).

## Results

### 46,XY DSD cases with uninformative exome sequencing

As previously described, we have performed exome sequencing on a cohort of 40 individuals diagnosed with 46,XY DSD [22]. To identify the disease-causing mutations, a DSD-specific gene list (published elsewhere [17]) was used for variant filtration. Exome sequencing was not able to identify the genetic diagnosis in > 50% of cases. To address this issue, we compiled a cohort of 32 DSD cases with uninformative exome and 46,XY karyotype for further investigation (Table 1) (this new cohort includes 21 unresolved cases from [22] and additional 11 cases with uninformative exomes enrolled since). As evident from Table 1, the range of associated clinical features was wide, which is a typical characteristic of DSD presentation. Patients could be grouped into four categories based on the appearance of the external genitalia and gonadal development: (1) 46,XY women with gonadal dysgenesis (GD), when gonadal phenotype had been ascertained by the clinical team; (2) 46,XY females; (3) 46,XY with ambiguous genitalia (and unknown sex of rearing at the time of enrollment); and (4) 46,XY males, with hypogonadism.

**Table 1:** Cohort of 46,XY DSD cases with uninformative exome sequencing

Patient ID	Category	DSD category	Clinical features
RDSD002	1	46,XY female, CGD	–
RDSD003	1	46,XY female, PGD	No uterus; Fallopian tubes present; short vagina; very low T and undetectable estradiol; gonads not found
RDSD004	1	46,XY female, GD	–
RDSD006	2	46,XY female	Amelia (missing limbs)
RDSD007	1	46,XY female, GD	Adrenal rests
RDSD010	2	46,XY female	Clitoromegaly
RDSD011	2	46,XY female	Short stature
RDSD012	2	46,XY female	Kidney disease; possible Denys-Drash syndrome
RDSD013	1	46,XY female, CGD	Normal uterus and Fallopian tubes; streak gonads
RDSD018	3	46,XY ambiguous genitalia	Partial fusion of labioscrotal folds; small phallus; penoscrotal hypospadias
RDSD020	3	46,XY ambiguous genitalia	Developmental delay; agenesis of corpus callosum
RDSD021	3	46,XY ambiguous genitalia	Adrenal hypoplasia congenita; dysmorphic features
RDSD022	3	46,XY ambiguous genitalia	Microcephaly; intestinal dysmotility; optic nerve hypoplasia
RDSD025	4	46,XY male, micropenis/cryptochidism	Severe growth and developmental retardation; testes not seen by ultrasound
CDSD029	4	46,XY male, hypospadias	–
CDSD030	2	46,XY female	Large clitoris; no uterus or vaginal opening; inguinal testes
CDSD031	3	46,XY ambiguous genitalia, CGD	Abdominal gonads with no oocytes; no seminiferous tubules; no clitoromegaly; posterior fusion of labia; urogenital sinus
CDSD032	2	46,XY female	Inguinal testes w/ immature seminiferous tubules; no uterus or Fallopian tubes; deafness; impaired cognition
CDSD034	3	46,XY ambiguous genitalia	Undescended testes; bifid scrotum; hypospadias
CDSD036	3	46,XY ambiguous genitalia	Bilateral descended testes; midshaft hypospadias; chordee
CDSD039	4	46,XY male, micropenis	No uterus or ovaries per ultrasound; ambiguous genitalia; undervirilization
RDSD041	2	46,XY female	Complete androgen insensitivity syndrome
RDSD042	4	46,XY male, hypospadias	–
RDSD043	1	46,XY female, GD	–
RDSD044	4	46,XY male, anorchia	Congenital bilateral anorchia; fully formed scrotum; definite penis (mildly shortened); no hypospadias; responsive to testosterone
RDSD045	4	46,XY male, hypospadias/cryptorchidism	Azoospermia; high T levels
RDSD046	2	46,XY female	Multiple congenital anomalies; no uterus; abdominal gonads—testes
RDSD047	4	46,XY male, microphallus	Hypogonadism; hypospadias
RDSD048	4	46,XY male, micropenis	–
RDSD049	4	46,XY male, hypospadias	–
CDSD050	4	46,XY male, hypospadias	Chordee; bifid scrotum; cryptorchidism
CDSD051	2	46,XY female	Growth delay; short stature

Anatomical description follows the standardized nomenclature in Hennekam et al. [55], except when only historical description was available in patient’s file. Patient IDs refer to cases enrolled for research purposes (RDSD) or enrolled through the UCLA clinical genomic center (CDSD). Numbering is not consecutive to maintain consistency with the numbering in Baxter et al. [22] for patients who are in both cohorts

*CGD/PGD* complete/partial gonadal dysgenesis

### C57BL/6J-Y *poschiavinus* mice as a model for 46,XY gonadal dysgenesis

In cases where no pathogenic variant was found by exome sequencing, we identified many VUS outside of the DSD clinical gene list. To investigate the relevance of these VUS in regard to patients’ phenotype, we utilized a powerful mouse model for studying undervirilization in human 46,XY individuals. In this model, the presence of a Y chromosome originating from a *M. domesticus poschiavinus* strain (Y^*POS*^) on a C57BL/6J (B6) background (B6-Y^*POS*^), an inbred laboratory strain that normally carries a *M. musculus* Y chromosome, results in disrupted testicular development and female genital phenotype [38].

**Fig 1:**
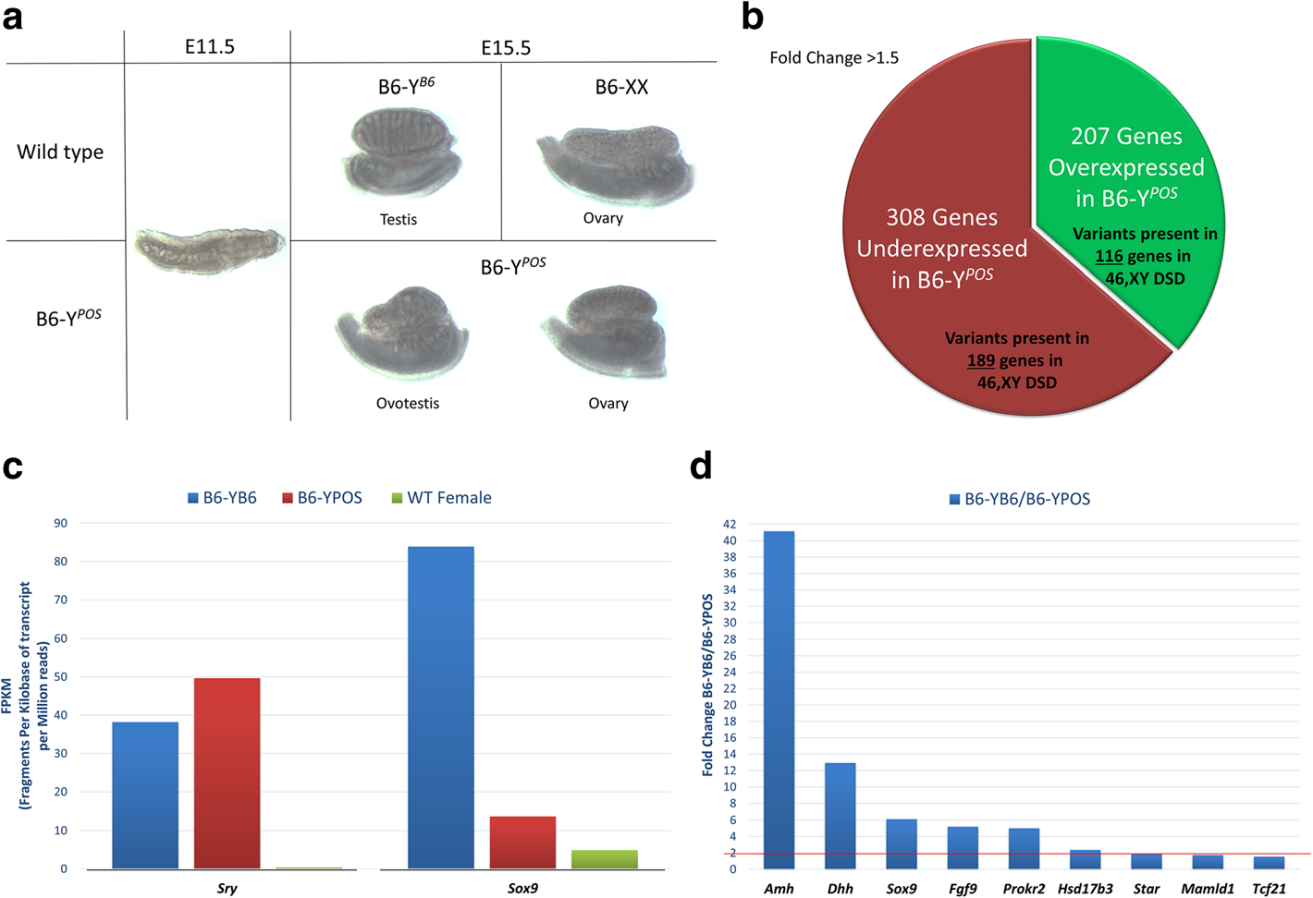
C57BL/6J-*Y poschiavinus* mice — a model for 46,XY DSD with gonadal dysgenesis. **a** The morphology of gonadal development in mice shown at embryonic day E11.5 (when it is still capable of giving rise to both testes and ovaries) and at E15.5. Top panel: testicular development in wild-type B6-Y^*B6*^ male and ovarian development in WT B6-XX female. Bottom panel: B6-Y^*POS*^ males show development of ovotestis (left) or ovary (right). **b** Pie chart representing the number of differentially expressed genes between WT B6-Y^*B6*^ and undervirilized B6-Y^*POS*^ male gonads at E11.5, as detected by RNASeq. Variants were found in exomes of the cohort of patients with 46,XY DSD in 189 of the 308 underexpressed and 116 of the 207 overexpressed genes. **c** Expression (shown as fragments per kilobase of transcript per million reads, FPKM) of the two major sex-determining genes *Sry* and *Sox9*. *Sry* expression was present in both B6-Y^*B6*^ and B6-Y^*POS*^ males. However, expression of *Sox9* was dramatically lower in B6-Y^*POS*^ males (as expected [41]); as a consequence, expression of some of the candidate genes for 46,XY DSD may be *Sox9*-dependent. **d** Expression values (shown as fold change differences between B6-Y^*B6*^ and B6-Y^*POS*^ males) for the 9 genes present in the primary gene list used for exome variant filtration that are also downregulated in B6-Y^*POS*^ males. The red line represents a 2-fold cutoff

This inherited phenomenon has been extensively studied in the B6-Y^*POS*^ animals. The failure to develop testes stems from the inability of the *Sry*^*POS*^ gene to initiate normal testicular development when B6 autosomal and/or X-linked factors are present. Virtually all B6-Y^*POS*^ animals develop some ovarian tissue; half develop exclusively ovarian tissue, classified as completely sex-reversed; and the remainder develop both ovarian and testicular tissue, classified as partially sex-reversed (gonad morphology shown in Fig. 1a). The B6-Y^*POS*^ mice represent a good model for studying 46,XY DSD with gonadal dysgenesis because of the overlap of the major phenotypic features that are present in both humans and mice such as normal physical appearance without clinical findings including major organs other than the reproductive system, normal karyotype, external genitalia that range from ambiguous to typical female-like, internal genitalia ranging from absent Müllerian structures to presence of a uterus, and abnormal gonadal development characterized as dysgenetic testes, streak, or ovotestes.

In the embryonic mouse gonad, *Sry* is normally expressed in a dynamic wave (central to distal) between E10.5 and E12.5 in the XY genital ridge, with peak *Sry* expression occurring in normal XY^*B6*^ genital ridges at ~E11.5, i.e., at the 16–18 tail somite stage of development, which is followed by the upregulation of *Sox9* [39, 40]. In contrast, expression of the *Sry*^*POS*^ gene peaks 10 to 14 h later in the genital ridges of B6-Y^*POS*^ fetuses [41]. We hypothesized that abnormal gonadal expression of specific genes in B6-Y^*POS*^ males, after the surge of *Sry* during gonadal development, would correlate with the genes in which VUS were identified in 46,XY DSD patients by exome sequencing.

### Gene expression differences between B6-Y^*B6*^ and of B6-Y^*POS*^ males

Since all of the 46,XY DSD patients in the cohort carried a functional *SRY* gene, it was important to perform gene expression analysis in the animal model after the peak of *Sry* expression for optimal comparability. To achieve this, gonadal tissue from WT B6-Y^*B6*^ and undervirilized B6-Y^*POS*^ males at embryonic day E11.5, specifically at 21 tail somites (a time point when the surge of *Sry* gene was complete in both B6-Y^*B6*^ and B6-Y^*POS*^ males), was collected to perform RNA sequencing for assessment of differential gene expression.

Using this method, we identified 515 genes that were differentially expressed between B6-Y^*B6*^ and B6-Y^*POS*^ males with a fold change greater than 1.5. Out of these 515 genes, 308 were underexpressed and 207 were overexpressed in B6-Y^*POS*^ males (Fig. 1b; Additional file 2: Table S2). To validate the integrity of the method and make sure that the correct tissue was dissected at the correct embryonic stages, we first looked at the expression levels of two important genes involved in testicular development, *Sry* and *Sox9.* High *Sry* and low *Sox9* expression levels in B6-Y^*POS*^ males indicated the correct timing of embryonic development (Fig. 1c), expression of which coincided with a previous publication [41]. Second, we verified which genes in our DSD gene list used for exome variant filtering were present in the B6-Y^*B6*^/B6-Y^*POS*^ differentially expressed gene list. The comparison of the two lists revealed that 21 genes were in common: 15 underexpressed and 6 overexpressed (Additional file 2: Table S2). In our previous cohort [22], out of these 21 genes, 3 (*HSD17B3* (hydroxysteroid 17-beta dehydrogenase 3), *STAR* (steroidogenic acute regulatory protein), *FGFR2*) contained a pathogenic variant identified by exome sequencing, explaining a total of 5 cases, and 2 (*DHH* (desert hedgehog), *MAMLD1* (mastermind-like domain-containing 1)) contained a variant that was reported to the clinician to orient further endocrine or imaging testing toward a definitive diagnosis. Cumulatively, these findings indicate that the gene expression analyses were carried out in a correct tissue type, at the correct developmental time point, and that the differentially expressed genes between B6-Y^*B6*^ and B6-Y^*POS*^ males may potentially be important in sex development.

### Filtering of VUS in 46,XY DSD cases using the B6-Y^*POS*^ gene list

On average, exome sequencing identifies ~ 21,000 variants per single case [23]. Since DSDs are rare conditions, all variants identified in exome with a minor allele frequency (MAF) of more than 1% in the population were excluded. The variants remaining after the MAF cutoff were classified as variants of unknown significance. The number of genes with a VUS in each case ranged from 30 to 1100 with an average of approximately 730 genes per case. The gene list generated via expression studies in B6-Y^*B6*^/B6-Y^*POS*^ mice, consisting of 515 genes, was used to filter the list of VUS-containing human genes identified by exome sequencing. The comparison of two lists identified 305 (189 underexpressed and 116 overexpressed in B6-Y^*POS*^) genes that were both differentially expressed in B6-Y^*POS*^ males and contained a VUS in the 46,XY DSD cohort with an uninformative exome (Fig. 1b).

All these genes are known to be expressed in the developing gonad at the time of sex determination (e.g., the method used to identify these genes intrinsically already ensures that all those genes are expressed in the relevant tissue (gonad) at the relevant developmental time). In order to increase the probability of identifying relevant candidate genes involved in 46,XY DSD pathogenesis, we further queried if the differentially expressed genes from the mouse model (all 515 genes) were involved in any known biological processes. Gene Ontology Consortium (GOC) [42] enrichment analysis confirmed that genes underexpressed in B6-Y^*POS*^ males were indeed enriched in biological processes known to control multicellular organism and anatomical structure development, including male reproductive development (Additional file 3: Table S3). Understanding the relevance of the genes that were overexpressed in B6-Y^*POS*^ males was less straightforward. These genes were enriched in only two biological processes: response to extracellular stimulus and epithelial cell differentiation. Both of these categories had a high *P* value indicating that many genes in the overexpressed category are not associated with any known biological processes at this time. In addition, all of the pathogenic variants identified in our previous 46,XY DSD cohort [22] were in the underexpressed category of genes, indicating that they need to be expressed at higher levels in the developing gonad for proper testicular formation.

Based on these findings, we focused on variants identified in genes underexpressed in B6-Y^*POS*^ males whose higher expression in WT males correlated with normal male sex development. To choose a fold change cutoff, we looked at fold change differences in expression between B6-Y^*B6*^ and B6-Y^*POS*^ males for genes present in our clinical primary gene list. We found that the majority of the genes have an expression that is 2-fold higher in WT males compared in B6-Y^*POS*^ males (Fig. 1d). To make the analysis more stringent and improve the confidence of identifying true candidate genes involved in male sex development, we therefore increased the fold change cutoff in expression between the B6-Y^*B6*^ and B6-Y^*POS*^ males from 1.5 to 2. This change decreased the number of underexpressed genes from 189 to 53. Additional filtering was performed based on variant frequency (variants with MAF close to or below 0.1%), amino acid conservation (variants in highly conserved residues across multiple species were given preference), number of variants contained in a gene across the cohort, in silico predictions for pathogenicity (preference was given to the variants predicted to be deleterious or damaging), availability of literature (some weight was given to genes with known functions), and gonadal cell-specific expressivity using GenitoUrinary Developmental Molecular Anatomy Project (GUDMAP) data [43] (preference was given to genes expressed in male-typical cells).

**Fig. 2:**
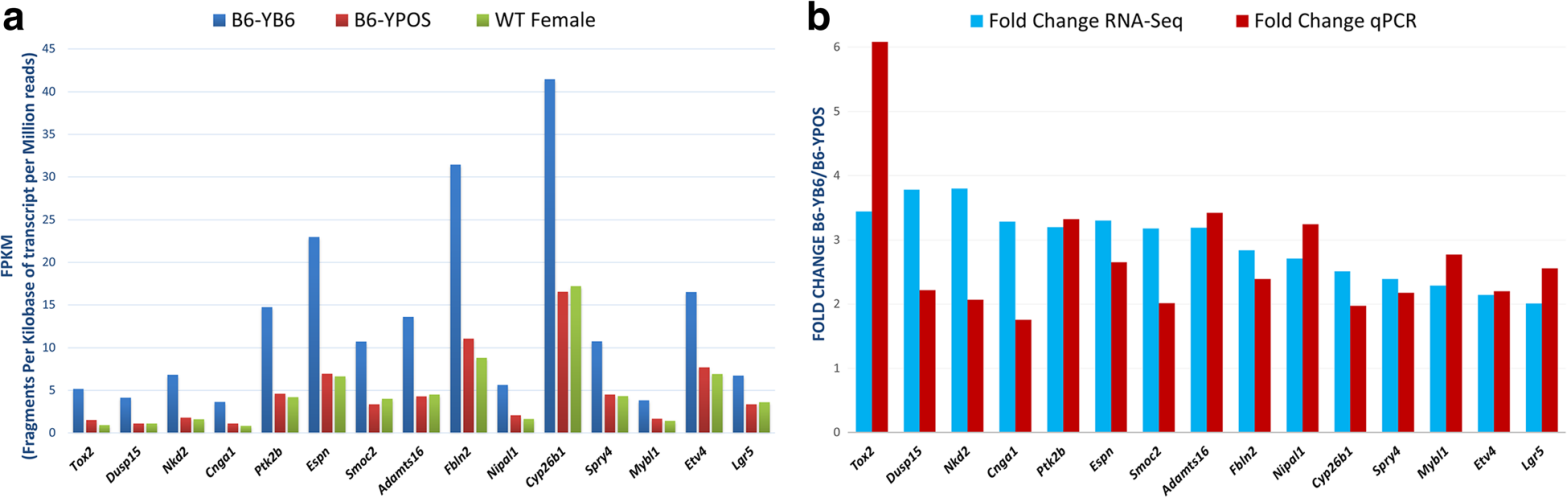
Gonadal expression of 15 novel candidate genes in the B6-Y^*POS*^ mouse model. **a** Gene expression differences in candidate genes between B6-Y^*B6*^ (blue) males, B6-Y^*POS*^ (red) males, and WT B6 females (green). The expression values, as measured by RNASeq, are shown in FPKM values (fragments per kilobase of transcript per million reads). Data generated from gonads dissected at E11.5. **b** Expression values are shown as fold change differences between B6-Y^*B6*^ and B6-Y^*POS*^ males using RNA-Seq data (blue) and qPCR data (red). Both methods show similar direction of gene expression in B6-Y^*B6*^ and B6-Y^*POS*^ males

Using the abovementioned filtering criteria, we identified 15 novel candidate sex developmental genes, variants in which may be involved in 46,XY DSD pathogenesis. The list of VUS identified in the 46,XY cohort is shown in Table 2. The relative expressions of these genes in B6-Y^*B6*^, B6-Y^*POS*^, and WT females are shown in Fig. 2a. The expression changes of all 15 genes were confirmed using quantitative real-time PCR (Fig. 2b).

**Table 2:** List of VUS in candidate genes found in the cohort of 32 46,XY DSD patients

Gene	DSD case ID	Zygosity	HGVSc	HGVSp	MAF gnomAD (%)
TOX2	RDSD021	Het	c.319G>A	p.Gly107Ser	0
	CDSD036	Cmpd Het	c.448A>G	p.Met150Val	0
	CDSD036	Cmpd Het	c.1201C>G	p.Pro401Ala	0
	CDSD036	Cmpd Het	c.1122_1124dupGCC	p.Pro376dup	0
DUSP15	RDSD020	Het	c.563G>C	p.Arg188Pro	0.002
NKD2	RDSD003	Het	c.1151G>A	p.Arg384Gln	0.001
CNGA1	CDSD030	Het	c.1478G>A	p.Arg493Gln	0.09
	RDSD022	Het	c.398G>T	p.Gly133Val	0.03
PTK2B	RDSD011	Het	c.1799G>A	p.Arg600Gln	0.0008
ESPN	RDSD044	Het	c.2230G>A	p.Asp744Asn	0.02
SMOC2	CDSD030	Het	c.1276G>A	p.Val426Met	0.3
ADAMTS16	RDSD013	Het	c.2200G>A	p.Val734Ile	0.8
	RDSD002	Het	c.298C>T	p.Arg100Trp	0.1
	RDSD022	Het	c.1405T>G	p.Phe469Val	0.02
FBLN2	CDSD030	Het	c.1486G>A	p.Ala496Thr	0.033
	CDSD029	Het	c.3605C>G	p.Ala1202Gly	0.004
NIPAL1	RDSD003	Het	c.1207A>G	p.Thr403Ala	0.1
	CDSD031	Het	c.31G>A	p.Glu11Lys	0
CYP26B1	CDSD032	Het	c.805C>G	p.Leu269Val	0.008
SPRY4	CDSD039	Het	c.446C>G	p.Pro149Arg	0.0004
MYBL1	RDSD004	Het	c.754T>A	p.Phe252Ile	0.05
	CDSD029	Het	c.1832G>C	p.Ser611Thr	0.0008
	RDSD049	Het	c.936T>A	p.Asn312Lys	0.03
ETV4	RDSD006	Het	c.523C>A	p.His175Asn	0.1
LGR5	RDSD007	Het	c.1834G>A	p.Val612Met	0.004
	RDSD020	Het	c.2341C>G	p.Pro781Ala	0.8
	RDSD048	Het	c.2537C>A	p.Thr846Asn	0

*Het* heterozygous, *Cmpd het* compound heterozygous, *HGVSc* Human Genome Variation Society coding sequence location, *HGVSp* Human Genome Variation Society protein sequence location, *MAF* minor allele frequency, *gnomAD* genome Aggregation Database

### Expression of the novel candidate genes is *Sox9*-dependent

**Fig. 3:**
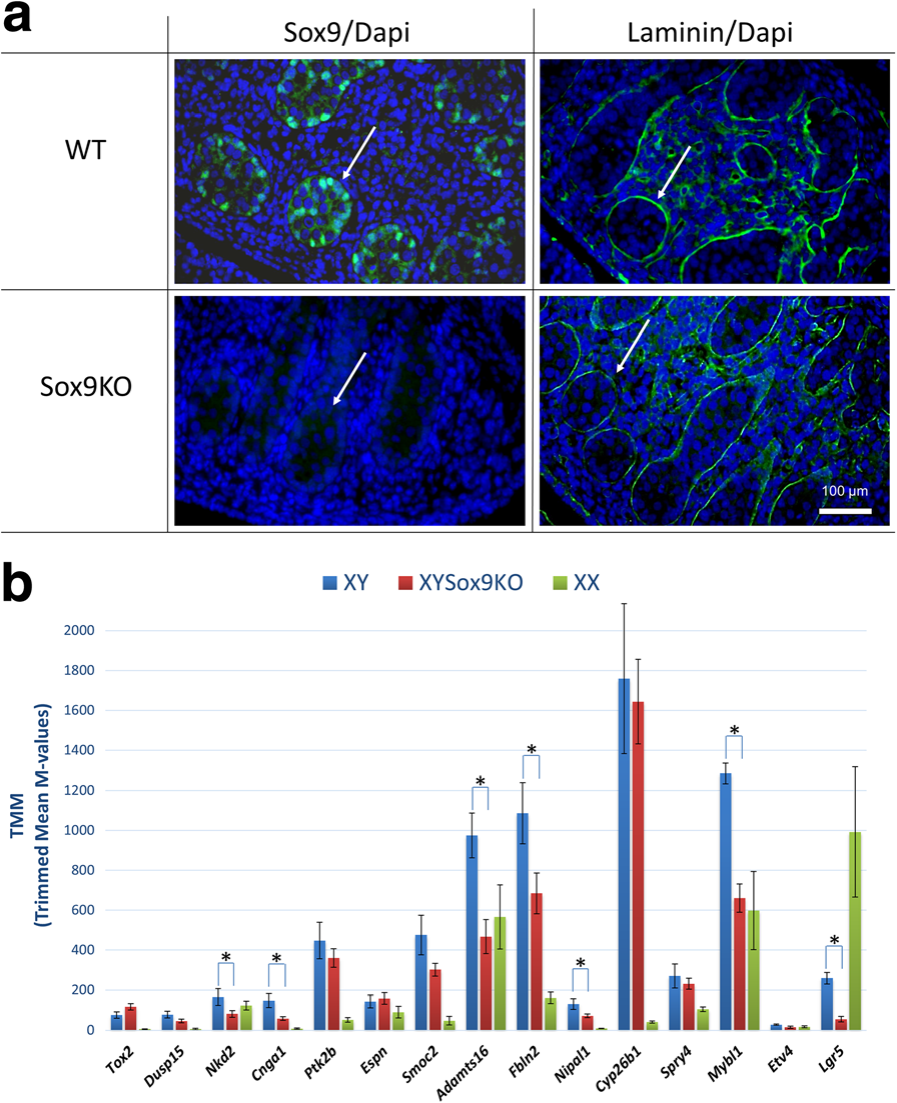
Expression of the novel candidate genes in *AmhCre Sox9floxflox* XY gonads. **a** Immunofluorescence of the wild-type and *Sox9* knockout gonad at E13.5. Sox9 protein is lost from the testicular cords (white arrows) in the *Amh-Cre Sox9floxflox* mice yet the testicular cords remain intact, as shown by the laminin stain. Sox9/Laminin is shown in green, and nuclei stained with DAPI are shown in blue. **b** Expression levels of candidate genes in *AmhCre Sox9floxflox* XY gonads (red) and WT B6 XY gonads (blue). Expression in WT B6 female gonads is also shown (XX in green). The expression values are shown in TMM values (trimmed mean of *M* values). RNA-seq was done *n* = 3 with six pooled E13.5 gonads in each sample. Error bars represent standard error of the mean. Asterisks indicate significantly differentially expressed genes based on an adjusted *P* value < 0.05

The time point chosen for our gene expression analysis was such that the *Sry* gene expression was similar between B6-Y^*B6*^ and B6-Y^*POS*^ males. At that time, the downstream target of *Sry,* *Sox9* was significantly decreased in B6-Y^*POS*^ males (Fig. 1c), as previously described [41]. In order to identify if *Sox9* had any effect on expression of the candidate genes, we used the *Amh-Cre Sox9flox/flox* mouse model where *Sox9* expression is suppressed in Sertoli cells [44]. By E13.5, Sox9 protein is completely absent (Fig. 3a), and these mice show postnatal fertility defects [44]. Earlier *Sox9* knockout models result in complete sex reversal (XY with ovaries) [45, 46] or embryonic lethality [47]; neither situation sheds light on *Sox9* target genes during sex determination. The *Amh-Cre Sox9flox/flox* mouse model allows the examination of *Sox9* loss in an intact Sertoli cell environment.

**Fig. 4:**
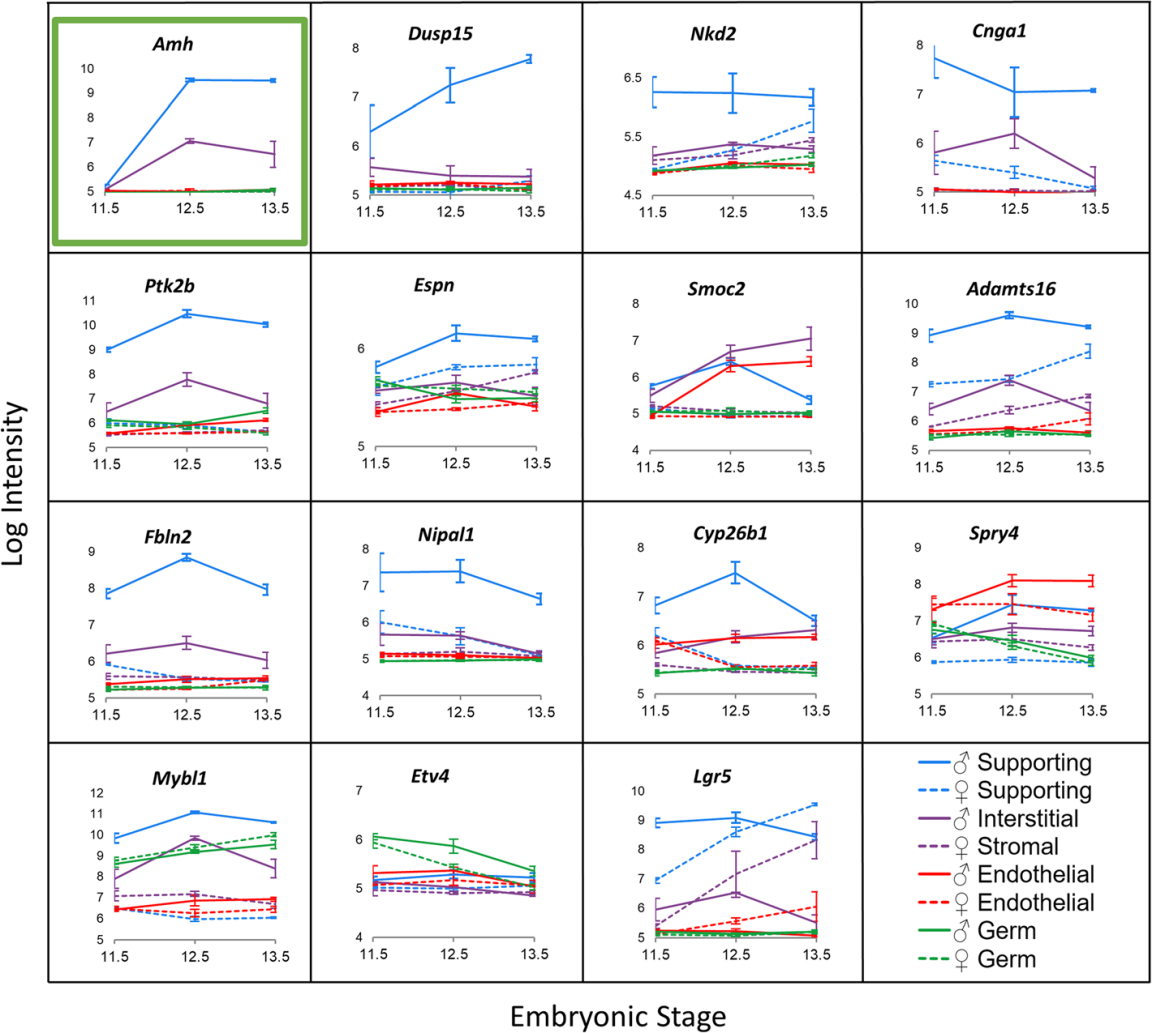
Profiles of candidate gene expression in the gonad at different sex developmental stages. Candidate gene profile graphs were generated from the microarray performed by Jameson et al. [42] where gene expression was profiled in each cell population of the gonad at E11.5, E12.5, and E13.5. Similar to the *Sox9* target genes *Amh* and *Ptgds* (only *Amh* is shown—outlined in green), the new candidate genes show strong expression in the male supporting lineage (solid blue line) compared to the female (dotted blue line). There was no information available in the microarray data for *Tox2*

Performing gene expression analysis via RNA sequencing in mice with suppressed *Sox9* expression showed that 13 of the novel candidate genes for 46,XY DSD underexpressed in B6-Y^*POS*^ males, were also underexpressed in *Sox9* knockout male gonads with 7 being significantly different (Fig. 3b). This finding suggests that *Sox9* may be upstream of some of the novel candidate genes for 46,XY DSD. In addition, the profiles of gonadal gene expressions from GUDMAP reveal that in almost all cases, the patterns of gene expression are similar to bona fide target genes of *Sox9* such as *Amh* (anti-Müllerian hormone) and *Ptgds* (prostaglandin D2 synthase) [42, 48, 49]. The target gene expression is higher in the male supporting cells (Sertoli) than in the female supporting cells (granulosa) (Fig. 4). The rest of the genes may be regulated by other transcription factors such as *Nr5a1*, which is a known regulator of *Cyp26b1* (cytochrome P450 family 26 subfamily B member 1) [50]. Collectively, our results show that variants in the candidate genes such as the ones we have identified in the 46,XY DSD cases (Table 2) may be responsible for the patient’s phenotype.

## Discussion

The use of the undervirilized B6-Y^*POS*^ mice as a model for 46,XY DSD in humans provides valuable screening information toward the identification of novel genes involved in male sex development, mutations in which could lead to anomalies in gonadal development in 46,XY patients with DSD. All of the identified candidate genes are expressed in the developing mouse gonad at the relevant time for sex determination and, as we have shown (Fig. 3a, b), the expression of many of these genes may be *Sox9-*dependent. However, when studying complex disorders such as DSD, it is important to note that even though the mouse models used here are extremely beneficial for identification of the underlying genetic cause in humans, they still do not provide the full spectrum of gene expression/interactions that occur during human sex development.

Mutations in the novel candidate genes identified via the Y^*POS*^ mouse model are likely to be causative. For example, one of the candidate genes *Adamts16* (A disintegrin and metalloproteinase with thrombospondin type 1 motif, 16) has been shown to be co-expressed with the known DSD gene *Wt1* (Wilms tumor 1) in embryonic gonads, adult testes, and spermatids [38]. Moreover, targeted disruption of *Adamts16* in rats results in cryptorchidism and sterility [51]. In our 46,XY DSD cohort, we identified three heterozygous variants in this gene (Table 2). Patients RDSD013 and RDSD002, both 46,XY women with complete gonadal dysgenesis, had a missense variant leading to amino acid change at positions p.Val734Ile and p.Arg100Trp respectively. These changes were located in the propeptide or cysteine-rich domain of the ADAMTS16 protein and may prevent expression or proper folding of the protein. The third missense variant (p.Phe469Val) in patient RDSD022 (46,XY, with ambiguous genitalia) was located in the peptidase domain of the protein and predicted damaging by in silico tools suggesting a possible impairment of the enzymatic function of ADAMTS16.

**Fig. 5:**
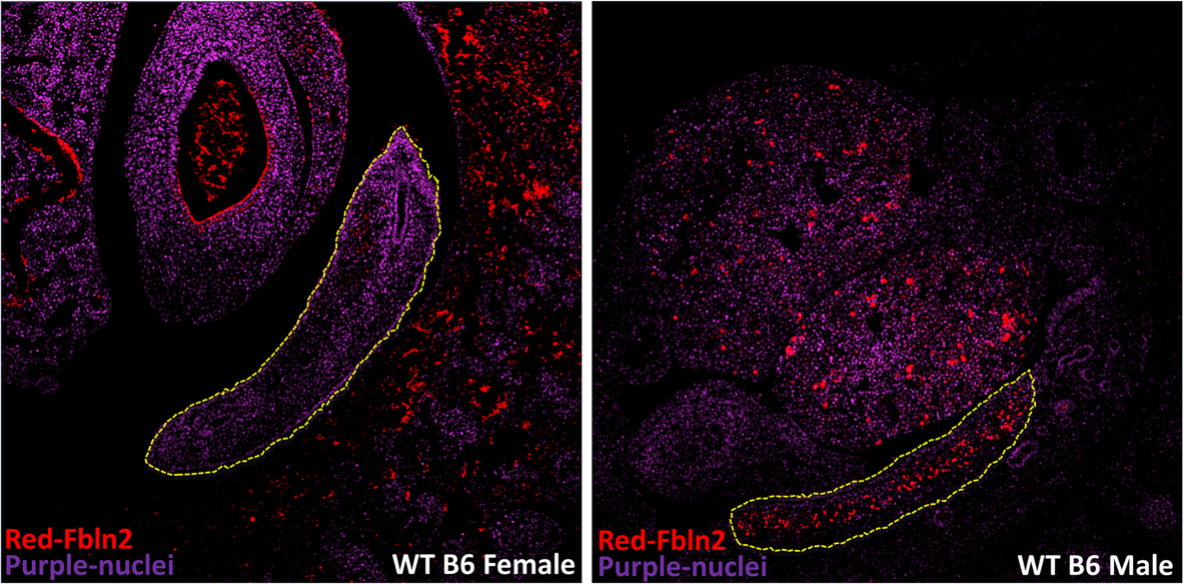
Fbln2 protein expression in WT B6 females and males at E12.5 by immunohistochemistry. Section of WT B6 female and male embryos at E12.5 stained for Fbln2 (red) and cell nuclei (purple). Fbln2 is expressed in a sexually dimorphic pattern as no expression is present in WT B6 female (left), whereas the expression in WT B6 male is high. The gonads are encircled by yellow dashed lines

We have also identified two rare variants (p.Ala496Thr; p.Ala1202Gly) in the *FBLN2* (fibulin 2) gene in two cases with different phenotypes: 46,XY female with inguinal testes/enlarged clitoris and 46,XY male with hypospadias. Additional rare *FBLN2* variants were present in six other unrelated cases with previously identified genetic diagnosis (i.e., each with a pathogenic variant identified in a known DSD gene). This suggest that variants in *FBLN2* are overrepresented in DSD population and may act as modifiers of the phenotype. We (Fig. 5) and others [52] show that *Fbln2* is expressed in a sexually dimorphic pattern in the developing gonad. Immunohistochemical staining at E12.5 indicated that WT B6 females have virtually no Fbln2 expression in the developing ovaries (Fig. 5, left panel), whereas WT B6 males (right panel) have very high expression in the developing testes suggesting an important role of Fbln2 in sexual dimorphism. *FBLN2* has been proposed as a candidate gene for 46,XY DSD in an unpublished meeting abstract (K. MacElreavey, personal communication).

We identified a single variant, predicted to be damaging by in silico tools, in the *SPRY4* (sprouty RTK signaling antagonist 4) gene in a 46,XY male patient (CDSD039) with hypogonadism. *SPRY4* variants have been found in a cohort of patients presenting with hypogonadotropic hypogonadism with or without anosmia (HH17, OMIM #615266) [53]. These genes are believed to be functioning in an oligogenic model, with variants in several genes possibly needed for phenotypic expression. Variants in *SPRY4* have been found in association with variants in *FGFR1* (fibroblast growth factor receptor 1) (HH2, OMIM #147950) and *DUSP6* (dual specificity phosphatase 6) (HH19, OMIM #615269), the two other FGF signaling pathway components. An *FGFR2* missense mutation was reported in a 46,XY female DSD patient, for which a corresponding mouse model showed partial sex reversal with reduced *Spry4* (2-fold) and *Dusp6* expression (> 2-fold) [54]. We did not identify *FGFR1* or *DUSP6* variants in the exome of patient CDSD039 (which would have been diagnostic for this patient). However, *DUSP6* is present in the differentially expressed gene list (underexpressed in B6-Y^*POS*^ with a fold change of 1.7) and another gene coding for a dual-specificity phosphatase, *DUSP15* (dual specificity phosphatase 15), is in our final candidate gene list, with underexpression in B6-Y^*POS*^ (fold change > 2) and contains a VUS in one patient.

## Conclusions

Exome sequencing provides high-throughput genetic diagnostic capability that has become the core of modern clinical genetics. However, many variants identified by whole exome sequencing are uninterpretable clinically. The C57/BL6J-Y^*POS*^ model narrows the interpretive gap by correlating human sequence variants with transcriptome variation. This approach allowed the identification of 15 novel candidate genes for human 46,XY DSD.

## Additional files

Additional file 1: Table S1. Primer sets used for quantitative PCR validation. (DOCX 12 kb)

Additional file 2: Table S2. Genes differentially expressed between B6-Y^*B6*^ and B6-Y^*POS*^ males. All 515 differentially expressed genes (column 1) either underexpressed or overexpressed (column 3) in B6-Y^*POS*^ males with corresponding fold change difference (column 2) are shown. The table also contains the DSD-specific gene list used to filter exome variants (column 4) as well as which genes are common between two lists (column 5). (XLSX 30 kb)

Additional file 3: Table S3. Biological processes in which differentially expressed genes are involved. The list of 515 genes found to be differentially expressed between B6-Y^*POS*^ and WT male embryonic gonads was analyzed using the Gene Ontology Consortium functional annotation software. The categories of Gene Ontology biological processes are shown in column 1. *P* value (column 5) is defined as the probability of seeing the indicated number of genes from the custom list (column 4) in the GO term gene list (column 3), given the total number of annotated genes in the whole genome. (DOCX 12 kb)

## References

[1] Koopman P, Sinclair A, Lovell-Badge R (2016). Of sex and determination: marking 25 years of Randy, the sex-reversed mouse. Development.

[2] Eggers S, Ohnesorg T, Sinclair A (2014). Genetic regulation of mammalian gonad development. Nat Rev Endocrinol.

[3] Ahmed SF, Hughes IA (2002). The genetics of male undermasculinization. Clin Endocrinol.

[4] Zhao F, Franco HL, Rodriguez KF, Brown PR, Tsai MJ, Tsai SY (2017). Elimination of the male reproductive tract in the female embryo is promoted by COUP-TFII in mice. Science.

[5] Arboleda VA, Fleming AA, Vilain E. Disorders of sex development. In: Weiss RE, Refetoff S, editors. Genetic diagnosis of endocrine disorders. London: Academic Press; 2010. p. 227–43.

[6] Délot E, Vilain E. Disorders of sex development. In: Strauss JF, Barbieri RL, Gargiulo AR, editors. Yen & Jaffe’s reproductive endocrinology. Philadelphia: Elsevier; 2019.

[7] Ono M, Harley VR (2013). Disorders of sex development: new genes, new concepts. Nat Rev Endocrinol.

[8] Lee PA, Houk CP, Ahmed SF, Hughes IA (2006). Consensus statement on management of intersex disorders. International Consensus Conference on Intersex. Pediatrics.

[9] Lee PA, Nordenstrom A, Houk CP, Ahmed SF, Auchus R, Baratz A (2016). Global disorders of sex development update since 2006: perceptions, approach and care. Horm Res Paediatr.

[10] Sandberg DE, Gardner M, Cohen-Kettenis PT (2012). Psychological aspects of the treatment of patients with disorders of sex development. Semin Reprod Med.

[11] Warne GL (2008). Long-term outcome of disorders of sex development. Sex Dev.

[12] Delot EC, Vilain EJ. Nonsyndromic 46,XX testicular disorders of sex development. In: Pagon RA, et al., editors. GeneReviews(R). Seattle; 1993.

[13] Baetens D, Stoop H, Peelman F, Todeschini AL, Rosseel T, Coppieters F (2017). NR5A1 is a novel disease gene for 46,XX testicular and ovotesticular disorders of sex development. Genet Med.

[14] Bashamboo A, Donohoue PA, Vilain E, Rojo S, Calvel P, Seneviratne SN (2016). A recurrent p.Arg92Trp variant in steroidogenic factor-1 (NR5A1) can act as a molecular switch in human sex development. Hum Mol Genet.

[15] Chapman C, Cree L, Shelling AN (2015). The genetics of premature ovarian failure: current perspectives. Int J Womens Health.

[16] Caburet S, Arboleda VA, Llano E, Overbeek PA, Barbero JL, Oka K (2014). Mutant cohesin in premature ovarian failure. N Engl J Med.

[17] Barseghyan H, Delot E, Vilain E (2015). New genomic technologies: an aid for diagnosis of disorders of sex development. Horm Metab Res.

[18] Granados A, Alaniz VI, Mohnach L, Barseghyan H, Vilain E, Ostrer H (2017). MAP3K1-related gonadal dysgenesis: six new cases and review of the literature. Am J Med Genet C Semin Med Genet.

[19] Delot EC, Papp JC (2017). DSD-TRN Genetics Workgroup, Sandberg DE, Vilain E. Genetics of disorders of sex development: the DSD-TRN experience. Endocrinol Metab Clin N Am.

[20] Eggers S, Sadedin S, van den Bergen JA, Robevska G, Ohnesorg T, Hewitt J (2016). Disorders of sex development: insights from targeted gene sequencing of a large international patient cohort. Genome Biol.

[21] Kim JH, Kang E, Heo SH, Kim GH, Jang JH, Cho EH (2017). Diagnostic yield of targeted gene panel sequencing to identify the genetic etiology of disorders of sex development. Mol Cell Endocrinol.

[22] Baxter RM, Arboleda VA, Lee H, Barseghyan H, Adam MP, Fechner PY (2015). Exome sequencing for the diagnosis of 46,XY disorders of sex development. J Clin Endocrinol Metab.

[23] Lee H, Deignan JL, Dorrani N, Strom SP, Kantarci S, Quintero-Rivera F (2014). Clinical exome sequencing for genetic identification of rare Mendelian disorders. JAMA.

[24] Yang Y, Muzny DM, Reid JG, Bainbridge MN, Willis A, Ward PA (2013). Clinical whole-exome sequencing for the diagnosis of mendelian disorders. N Engl J Med.

[25] Arboleda VA, Fleming A, Barseghyan H, Delot E, Sinsheimer JS, Vilain E (2014). Regulation of sex determination in mice by a non-coding genomic region. Genetics.

[26] Umemura Y, Miyamoto R, Hashimoto R, Kinoshita K, Omotehara T, Nagahara D (2016). Ontogenic and morphological study of gonadal formation in genetically-modified sex reversal XY(POS) mice. J Vet Med Sci.

[27] Li H, Durbin R (2010). Fast and accurate long-read alignment with burrows-wheeler transform. Bioinformatics.

[28] Rehm HL, Bale SJ, Bayrak-Toydemir P, Berg JS, Brown KK, Deignan JL (2013). ACMG clinical laboratory standards for next-generation sequencing. Genet Med.

[29] Kumar P, Henikoff S, Ng PC (2009). Predicting the effects of coding non-synonymous variants on protein function using the SIFT algorithm. Nat Protoc.

[30] Adzhubei IA, Schmidt S, Peshkin L, Ramensky VE, Gerasimova A, Bork P (2010). A method and server for predicting damaging missense mutations. Nat Methods.

[31] Strom SP, Lee H, Das K, Vilain E, Nelson SF, Grody WW (2014). Assessing the necessity of confirmatory testing for exome-sequencing results in a clinical molecular diagnostic laboratory. Genet Med.

[32] Rahmoun M, Lavery R, Laurent-Chaballier S, Bellora N, Philip GK, Rossitto M (2017). In mammalian foetal testes, SOX9 regulates expression of its target genes by binding to genomic regions with conserved signatures. Nucleic Acids Res.

[33] Dobin A, Davis CA, Schlesinger F, Drenkow J, Zaleski C, Jha S (2013). STAR: ultrafast universal RNA-seq aligner. Bioinformatics.

[34] Trapnell C, Williams BA, Pertea G, Mortazavi A, Kwan G, van Baren MJ (2010). Transcript assembly and quantification by RNA-Seq reveals unannotated transcripts and isoform switching during cell differentiation. Nat Biotechnol.

[35] The Gene Ontology, C (2017). Expansion of the Gene Ontology knowledgebase and resources. Nucleic Acids Res.

[36] Robinson MD, Oshlack A (2010). A scaling normalization method for differential expression analysis of RNA-seq data. Genome Biol.

[37] Wilhelm D, Washburn LL, Truong V, Fellous M, Eicher EM, Koopman P (2009). Antagonism of the testis- and ovary-determining pathways during ovotestis development in mice. Mech Dev.

[38] Eicher EM, Washburn LL, Whitney JB, Morrow KE (1982). Mus poschiavinus Y chromosome in the C57BL/6J murine genome causes sex reversal. Science.

[39] Bullejos M, Koopman P (2001). Spatially dynamic expression of Sry in mouse genital ridges. Dev Dyn.

[40] Kobayashi A, Chang H, Chaboissier MC, Schedl A, Behringer RR (2005). Sox9 in testis determination. Ann N Y Acad Sci.

[41] Bullejos M, Koopman P (2005). Delayed Sry and Sox9 expression in developing mouse gonads underlies B6-Y(DOM) sex reversal. Dev Biol.

[42] Jameson SA, Natarajan A, Cool J, DeFalco T, Maatouk DM, Mork L (2012). Temporal transcriptional profiling of somatic and germ cells reveals biased lineage priming of sexual fate in the fetal mouse gonad. PLoS Genet.

[43] Harding SD, Armit C, Armstrong J, Brennan J, Cheng Y, Haggarty B (2011). The GUDMAP database—an online resource for genitourinary research. Development.

[44] Barrionuevo F, Georg I, Scherthan H, Lecureuil C, Guillou F, Wegner M (2009). Testis cord differentiation after the sex determination stage is independent of Sox9 but fails in the combined absence of Sox9 and Sox8. Dev Biol.

[45] Barrionuevo F, Bagheri-Fam S, Klattig J, Kist R, Taketo MM, Englert C (2006). Homozygous inactivation of Sox9 causes complete XY sex reversal in mice. Biol Reprod.

[46] Lavery R, Lardenois A, Ranc-Jianmotamedi F, Pauper E, Gregoire EP, Vigier C (2011). XY Sox9 embryonic loss-of-function mouse mutants show complete sex reversal and produce partially fertile XY oocytes. Dev Biol.

[47] Bi W, Huang W, Whitworth DJ, Deng JM, Zhang Z, Behringer RR (2001). Haploinsufficiency of Sox9 results in defective cartilage primordia and premature skeletal mineralization. Proc Natl Acad Sci U S A.

[48] Behringer RR, Finegold MJ, Cate RL (1994). Müllerian-inhibiting substance function during mammalian sexual development. Cell.

[49] Moniot B, Declosmenil F, Barrionuevo F, Scherer G, Aritake K, Malki S (2009). The PGD2 pathway, independently of FGF9, amplifies SOX9 activity in Sertoli cells during male sexual differentiation. Development.

[50] Kashimada K, Svingen T, Feng CW, Pelosi E, Bagheri-Fam S, Harley VR (2011). Antagonistic regulation of Cyp26b1 by transcription factors SOX9/SF1 and FOXL2 during gonadal development in mice. FASEB J.

[51] Abdul-Majeed S, Mell B, Nauli SM, Joe B (2014). Cryptorchidism and infertility in rats with targeted disruption of the Adamts16 locus. PLoS One.

[52] Bouma GJ, Hudson QJ, Washburn LL, Eicher EM (2010). New candidate genes identified for controlling mouse gonadal sex determination and the early stages of granulosa and Sertoli cell differentiation. Biol Reprod.

[53] Miraoui H, Dwyer AA, Sykiotis GP, Plummer L, Chung W, Feng B (2013). Mutations in FGF17, IL17RD, DUSP6, SPRY4, and FLRT3 are identified in individuals with congenital hypogonadotropic hypogonadism. Am J Hum Genet.

[54] Bagheri-Fam S, Ono M, Li L, Zhao L, Ryan J, Lai R (2015). FGFR2 mutation in 46,XY sex reversal with craniosynostosis. Hum Mol Genet.

[55] Hennekam RC, Allanson JE, Biesecker LG, Carey JC, Opitz JM, Vilain E (2013). Elements of morphology: standard terminology for the external genitalia. Am J Med Genet A.

