# Supplementary material for: Identification of novel candidate genes for 46,XY disorders of sex development (DSD) using a C57BL/6J-YPOS mouse model
Source: Biol Sex Differ. 2018 Jan 30;9:8. doi: 10.1186/s13293-018-0167-9 (PMC5789682; doi:10.1186/s13293-018-0167-9)
Supplement: Supplementary file 1 — Primer sets used for quantitative PCR validation. (DOCX 12 kb) [file 13293_2018_167_MOESM1_ESM.docx]

**Additional file 1: Table S1**

| Gene | Forward primer | Reverse primer | Annealing temperature  (in Celsius) |
| --- | --- | --- | --- |
| *Tox2* | GGCCTACGCTCTCTTCTTC | CCTCTTATACGCCTGTTTCTG | 60 |
| *Dusp15* | AACTTCATTGATGCCAAAGAC | GGTGATTCGTGGATAGAGATG | 61 |
| *Nkd2* | GTGGCAGAACAGAGATTGAG | TTGGGCTTCCTGCTGTAG | 60 |
| *Cnga1* | TCAACAACAGCAGCAACAAAG | TATCATCGGCCTTGCTCTTC | 64 |
| *Ptk2b* | TGGATGTGGAGAAGGAAGAC | TGATGATCTCCTGGATCTCTG | 61 |
| *Espn* | ATTACCCTGAGGGAGTGAATG | AAGGTACTTCGTCACTTCCAG | 60 |
| *Smoc2* | CAAATGGAAGACCCATCAG | CAGCATCATCTGCTTTCC | 60 |
| *Adamts16* | AACATGGTGTCTGCCTTATTC | CCCTGGCTGTTCATCTTC | 63 |
| *Fbln2* | GTGATCTTGATGGCTCCAC | CTGGGCTATCCTACAGATGTC | 60 |
| *Nipal1* | GGGTCAACTGTGATGGTTATC | AACGAACCCTGGATCTCTC | 61 |
| *Cyp26b1* | GTACCCAGGGCAAAGACTAC | GGTTCCATCCTTCAGCTC | 60 |
| *Spry4* | TGCAGCTCCTCAAAGACC | ATGACTGAGCTGGGATTCAC | 60 |
| *Mybl1* | GTCAGCCGAGAATGAAGTTAG | AGCTTCCAGGTTGAGGTG | 61 |
| *Etv4* | CAGGACCTCAGTCACTTCC | CGGTACCTGAGCTTCTGC | 62 |
| *Lgr5* | AACCTCCGATCTCTGAACTTAG | CGACAGGAGATTGGATGATAG | 63 |
