# Supplementary material for: Identification of novel candidate genes for 46,XY disorders of sex development (DSD) using a C57BL/6J-YPOS mouse model
Source: Biol Sex Differ. 2018 Jan 30;9:8. doi: 10.1186/s13293-018-0167-9 (PMC5789682; doi:10.1186/s13293-018-0167-9)
Supplement: Supplementary file 3 — Biological processes in which differentially expressed genes are involved. The list of 515 genes found to be differentially expressed between B6-YPOS and WT male embryonic gonads was analyzed using the Gene Ontology Consortium functional annotation software. The categories of Gene Ontology biological processes are shown in column 1. P value (column 5) is defined as the probability of seeing the indicated number of genes from the custom list (column 4) in the GO term gene list (column 3), given the total number of annotated genes in the whole genome. (DOCX 12 kb) [file 13293_2018_167_MOESM3_ESM.docx]

**Additional file 3: Table S3**

| Gene Ontology Biological Process | Expression in B6-Y*^POS^* vs. B6-Y*^B6^* Males | # of genes in the GO Term Reference Gene List | # of genes Test Gene List | P-value <0.05 |
| --- | --- | --- | --- | --- |
| Single-multicellular organism process (GO:0044707) | Underexpressed | 5417 | 151 | 4.49E-15 |
| System development (GO:0048731) | Underexpressed | 4042 | 125 | 1.45E-14 |
| Multicellular organism development (GO:0007275) | Underexpressed | 4640 | 136 | 1.61E-14 |
| Anatomical structure development (GO:0048856) | Underexpressed | 4986 | 139 | 4.32E-13 |
| Multicellular organismal process (GO:0032501) | Underexpressed | 6482 | 163 | 1.58E-12 |
| Reproductive system development (GO:0061458) | Underexpressed | 429 | 26 | 1.21E-05 |
| Male sex differentiation (GO:0046661) | Underexpressed | 161 | 15 | 1.86E-04 |
| Reproduction (GO:0000003) | Underexpressed | 1373 | 45 | 2.52E-03 |
| Male gonad development (GO:0008584) | Underexpressed | 139 | 12 | 1.05E-02 |
| Response to extracellular stimulus (GO:0009991) | Overexpressed | 488 | 18 | 3.08E-02 |
| Epithelial cell differentiation (GO:0030855) | Overexpressed | 503 | 18 | 4.65E-02 |
